# DPP‐4 inhibitor sitagliptin treatment results in altered myocardial metabolic proteome and oxidative phosphorylation in a swine model of chronic myocardial ischemia

**DOI:** 10.14814/phy2.15976

**Published:** 2024-03-12

**Authors:** Dwight D. Harris, Sharif A. Sabe, Mark Broadwin, Krishna Bellam, Cynthia M. Xu, Janelle W. Li, M. Ruhul Abid, Frank W. Sellke

**Affiliations:** ^1^ Division of Cardiothoracic Surgery, Department of Surgery Cardiovascular Research Center, Rhode Island Hospital, Alpert Medical School of Brown University Providence Rhode Island USA

**Keywords:** chronic myocardial ischemia, DPP‐4 inhibitor, oxidative phosphorylation, proteomics, sitagliptin

## Abstract

Small animal models have shown improved cardiac function with DPP‐4 inhibition, but many human studies have shown worse outcomes or no benefit. We seek to bridge the gap by studying the DPP‐4 inhibitor sitagliptin in a swine model of chronic myocardial ischemia using proteomic analysis. Thirteen Yorkshire swine underwent the placement of an ameroid constrictor on the left coronary circumflex artery to model chronic myocardial ischemia. Two weeks post‐op, swine received either sitagliptin 100 mg daily (SIT, *n* = 5) or no drug (CON, *n* = 8). After 5 weeks of treatment, swine underwent functional measurements and tissue harvest. In the SIT group compared to CON, there was a trend towards decreased cardiac index (*p* = 0.06). The non‐ischemic and ischemic myocardium had 396 and 166 significantly decreased proteins, respectively, in the SIT group compared to CON (all *p* < 0.01). This included proteins involved in fatty acid oxidation (FAO), myocardial contraction, and oxidative phosphorylation (OXPHOS). Sitagliptin treatment resulted in a trend towards decreased cardiac index and decreased expression of proteins involved in OXPHOS, FAO, and myocardial contraction in both ischemic and non‐ischemic swine myocardium. These metabolic and functional changes may provide some mechanistic evidence for outcomes seen in clinical studies.

## 1 INTRODUCTION

Chronic coronary artery disease (CAD) remains a leading cause of mortality worldwide (Brown et al., 2023). Medical therapy for patients with advanced CAD not amenable to surgical or percutaneous intervention is limited (Knuuti et al., 2020; Lassaletta et al., 2011). As a result, there is growing interest in the use of novel diabetic medications in the management of CAD. Several agents including sodium‐glucose transport protein 2 (SGLT‐2) inhibitors and glucagon‐like peptide 1 (GLP‐1) receptor agonists have shown significant promise in treating patients with CAD and heart failure in both pre‐clinical and clinical studies (Al Thani et al., 2023; Bhagavathula et al., 2021; McMurray et al., 2019; Sabe, Xu, et al., 2023; Wiviott et al., 2019). This growing body of evidence has resulted in guideline changes favoring the use of GLP‐1 agonist and SGLT‐2 inhibitors (*Circulation*, 2022; Knuuti et al., 2020). Interestingly, another class of antidiabetic medications, dipeptidyl peptidase 4 (DPP‐4) inhibitors have shown mixed results.

DPP‐4 inhibitors increase insulin sensitivity and release by preventing the breakdown of incretins such as GLP‐1 (Vella, 2012). The increase in GLP‐1 results in increased insulin release and inhibits the release of glucagon. Interestingly, GLP‐1 agonists and DPP‐4 inhibitors work at similar steps in the same pathway but have yielded different clinical results. In human studies, DPP‐4 inhibitors have shown mixed results with most studies showing no benefit from the use of DPP‐4 inhibitors (Gantz et al., 2017; Green et al., 2015; Rosenstock, Kahn, et al., 2019; Rosenstock, Perkovic, et al., 2019; White et al., 2013). Interestingly, the Savor‐TIMI 53 trial showed an increase in heart failure readmissions in the DPP‐4 group (Scirica et al., 2013). This finding has not been replicated in smaller clinical studies but has resulted in increased caution when prescribing DPP‐4 inhibitors (Patoulias et al., 2021).

Interestingly, there is a disconnect between animal studies of DPP‐4 inhibitors and the aforementioned clinical studies on the effects of DPP‐4 inhibitors in patients with CAD. Mouse studies have shown increased angiogenesis, decreased ischemic reperfusion injury, and decreased coronary atherosclerosis with DPP‐4 inhibitors (Abbas et al., 2018; Al‐Awar et al., 2018; Bradic et al., 2021; Fan et al., 2020; Khodeer et al., 2019; Zakaria et al., 2022). Other mouse studies modeling heart failure have shown increased cardiac function and decreased diastolic dysfunction with DPP‐4 inhibitors (Beraldo et al., 2019; Esposito et al., 2017). A swine study using heart rate‐induced heart failure showed increased stroke volume with DPP‐4 inhibitor treatment (Gomez et al., 2012). Thus, most animal trials have shown cardiovascular benefits with DPP4i, although one mouse study showed worsening heart failure with DPP‐4 inhibitor treatment (Shiraki et al., 2022). These results are in contrast to the lack of cardiovascular benefit with DPP‐4 inhibitors seen in clinical trials.

Given the disconnect between small animal and clinical studies, we set out to investigate the effects of DPP‐4 inhibitor sitagliptin using our established, clinically relevant swine model for chronic myocardial ischemia. We have previously shown improved myocardial perfusion, increased arteriolar collateralization, and activation of pro‐arteriogenic signaling pathways in swine treated with DPP‐4 inhibitors (Sabe, Harris, et al., 2023). However, there was no change in cardiac output, stroke volume, or ventricular stiffness, and the study was complicated by increased mortality in the sitagliptin group leaving questions about the safety of DPP‐4 inhibitors in chronic myocardial ischemia (Sabe, Harris, et al., 2023). We seek to better understand the mechanisms of DPP‐4 inhibitors in the ischemic myocardium by using proteomic analysis.

## 2 METHODS

### 2.1 Model

This study uses the cohort of 13 non‐randomized, non‐blinded swine previously described (Sabe, Harris, et al., 2023). Yorkshire swine (Cummings School of Veterinary Medicine of Tufts University Farm, North Grafton, MA, USA) fed a normal diet (Teklad Mini Pig Diet # 8753, Inotiv, West Lafayette, IN, USA) underwent left thoracotomy and ameroid constrictor (Research Instruments SW, Escondido, CA, USA) placement on the left coronary circumflex (LCx) at age 11 weeks. Two weeks after placement of the ameroid constrictors animals received either sitagliptin 100 mg daily (SIT, *n* = 5, Female = 1, Male = 4; #NDC 0006‐0277‐31, Merck & Co., Rahway, New Jersey, USA) or vehicle without drug (CON, *n* = 8, Female = 3, Male = 5). After 5 weeks of treatment, the swine underwent functional measurement and euthanasia for tissue collection.

### 2.2 Animal care

The protocol was approved by the Rhode Island Hospital Institutional Animal Care and Use Committee (IACUC) (Protocol #505821) (Sabe, Harris, et al., 2023). Animals were cared for in compliance with the Principles of Laboratory Animal Care and the Guide for the Care and Use of Laboratory Animals. All swine are house in rooms of three to allow for social interaction. The animals have continues access to water and have their bedding changed daily.

### 2.3 Ameroid constrictor placement

The anesthesia and preoperative care were performed as described in our prior publication (Sabe, Harris, et al., 2023). The swine was placed in a modified right lateral decubitus position. A left thoracotomy was performed at the second intercostal space. The pericardium was entered, and the left coronary circumflex was exposed with blunt and sharp dissection. The left coronary circumflex was circled with a vessel loop and occluded for 2 min while injecting 5 mL gold microspheres into the left atrium (# C‐10H10, BioPal, Worcester, MA, USA) to mark the area at risk (Sabe, Harris, et al., 2023). The ameroid constrictor was placed as proximal to the take‐off of the left circumflex as possible to create consistent areas of ischemia. The pericardium and chest were closed in layers, as previously described.

### 2.4 Pause in protocol

It should be noted that the protocol originally had 10 animals allotted to the sitagliptin group (Sabe, Harris, et al., 2023). As described in our prior paper, the mortality was 50% in the sitagliptin group (Sabe, Harris, et al., 2023). This was higher than the historic mortality of less than 20%. The IACUC at Rhode Island Hospital temporarily paused treatment in the remaining two of the animals for 2 weeks given the mortality. The study was allowed to finish the remaining two animals with a one‐week course prior to harvest (Sabe, Harris, et al., 2023).

### 2.5 Harvest and functional measurements

The anesthesia and preoperative care were performed as described (Sabe, Harris, et al., 2023). The swine was placed supine. A median sternotomy was performed, and the pericardium was dissected free for sternal adhesions. The pericardium was opened. The femoral artery was accessed in the right groin. One pressure catheter (#FDH‐5018B‐E345D, Transonic, Ithaca, New York, USA) was advanced into the aorta for the groin, and a pressure–volume catheter (#FDH‐5018B‐E345D, Transonic, Ithaca, New York, USA) was then inserted into the apex of the left ventricle as previously described (Sabe, Xu, et al., 2023). Hemodynamic data were recorded and analyzed as previously described with LabChart software (ADInstruments, Sydney, Australia) (Sabe, Xu, et al., 2023). Myocardial perfusion was calculated in our previous study using isotope‐labeled microspheres (# C‐10A10 and # C‐10E10, BioPal, Worcester, MA) as previously described (Sabe, Harris, et al., 2023). Data for the ischemic myocardial perfusion was obtained at rest and while pacing the heart at 150 beats per minute. One hundred and fifty beats per minute was chose as it is a rate we have previously used above the normal range of 80–120 to induce stress (Lassaletta et al., 2011; Sabe, Harris, et al., 2023). A glucose tolerance test was performed at the harvest. Each swine was given a 1 mL/kg bolus of a 50% dextrose solution at the start of the case. The blood glucose was measured at 30 and 60 min. After completion of functional measurements, the animal was euthanized by cardiectomy, and the heart was sectioned into 16 segments and snap frozen in liquid nitrogen for further analysis (Sabe, Harris, et al., 2023). Microsphere analysis was used to confirm the most ischemic (usually superior free wall area) and least ischemic (usually distal anterior area) left ventricular myocardial tissue for analysis.

### 2.6 Proteomics

The tissue samples were homogenized in lysis buffer T‐PER (#78510, ThermoFisher, Waltham, MA, USA) with a Halt Protease Inhibitor tablet (#A32953, ThermoFisher, Waltham, MA, USA) using a BeadBug homogenizer with 3 mm zirconium beads (#D1032‐30, Benchmark Scientific, Sayreville, NJ, USA). The supernatants were removed, and a BCA assay was performed to determine protein concentration. Aliquots of 100 μg were buffer exchanged via addition of 4x by volume chilled acetone (#A929‐1, ThermoFisher, Waltham, MA, USA). The samples were precipitated overnight at −20°C. The proteins were pelleted via centrifugation at 16,000 × *g* for 10 min. The protein pellets were rinsed with 200 μL of 90% chilled acetone and recollected via centrifugation at 16,000 × *g* for 5 min. The supernatants were discarded, and the protein pellet was air‐dried for 5 min. The samples were resuspended in 100 mM tetraethylammonium bromide (#90114, ThermoFisher, Waltham, MA, USA), reduced with 2.1 μL of 500 mM dithiothreitol (#171660050, ThermoFisher, Waltham, MA, USA) incubated for 45 min at 60°C, alkylated with 11.5 μL of 500 mM Indole‐3‐acetic acid (#122270250, ThermoFisher, Waltham, MA, USA) incubated at RT in the dark for 30 min, and digested overnight with 2.5 μL of 1 mg/mL Trypsin/Lys‐C (#A41007, ThermoFisher, Waltham, MA, USA) at 37°C. The samples final concentration was 1 mg/mL. Of each digest, 1 μg was labeled with the appropriate TMTpro reagent (ThermoFisher, Waltham, MA, USA). A TMT pro16 plex kit (A44521, ThermoFisher, Waltham, MA, USA) was supplemented with TMTpro 18 plex label 135 N (A52046, ThermoFisher, Waltham, MA, USA). Labeling reactions were incubated on the benchtop for 1 h and quenched with 1 μL of 5% hydroxylamine (#612‐122‐01‐4, Sigma Aldrich, Burlington, MA, USA) for 15 min. The labeled samples were multiplexed and dried via a vacufuge.

The samples were run on an Evosep One (Evosep, Odense, Denmark) nLC coupled online to a Bruker timsTOF HT mass spectrometer (Bruker Scientific LLC, Billerica, MA, USA). The samples were run on the Evosep 30 samples per day (SPD) gradient (Evosep, Odense, Denmark) on a PepSep Endurance column (15 cm × 150 mm, 1.9 μm) (1,893,471, PepSep, Odense, Denmark). The timsTOF was operated in PASEF mode with a scan range of 100–1700 m/z and a mobility range of 0.60–1.60 V·s/cm^3^. The ramp time and accumulation times were both set to 100.0 ms. The duty cycle was set to 100.0%, ramp rate to 9.42 Hz, and the MS averaging to 1. For the MS/MS parameters, the number of PASEF ramps was set to 10 with a total cycle time of 2.12 s. The charge minimum was set to 0, and the maximum to 5. The target intensity was set to 20,000, and the intensity threshold was set to 2500. The collision energies were set to 20.00 eV and CE #2 32.00 eV for 1/K0 0.60 and 59.00 eV, and CE #2 64.00 eV for 1.60 1/K0.

Protein identification and quantification analysis were done with PaSER (2023, v 3.0, Bruker Scientific LLC, Billerica, MA, USA). Quantitative data is reported only when a reporter ion was detected in all channels within a group. Mass spectra were streamed via the PaSER plugin directly from the timsTOF's acquisition control software to the PaSER workstation and were searched against Sus scrofaprotein database downloaded on 18 September 2022 plus sequences of known contaminants such as keratin and porcine trypsin concatenated to a decoy database in which the sequence for each entry in the original database was reversed. Search space included all fully−/half‐tryptic peptide candidates with two missed cleavages. Carbamidomethylation of cysteine, TMTpro of lysine and arginine, and TMTpro of the N‐termini were considered static modifications, and we require 1 peptides per protein and at least one tryptic terminus for each peptide identification. Additionally, the oxidation of methionine was considered as a variable modification and up to two variable modifications were allowed per peptide. TIMScore was appended to raw search results to use the peptide Collisional Cross Section during the validation process. These search results were validated, assembled, and filtered using the DTASelect program version 2.1 (Yates, La Jolla, CA, USA) with a false discovery rate (FDR) of 0.01; under such filtering conditions, the estimated false discovery rate was below ~1% at the protein level in all analysis with peptide FDR <0.01. There was a mass tolerance of 20.0 ppm and an isolation window of 0.8 m/z was applied.

Protein identification and quantification were done by PEAKS Studio (10.6 Build, Bioinformatics Solutions Inc., Waterloo, Ontario, Canada). One unique peptide was required for Protein Identification. For quantifications, only unique peptides that scored above a 1% FDR were used for protein quantification. The intensities of TMT reporter ions in this subset of peptides were summed to obtain the TMT intensities for the protein. PEAKS de novo workflow, database workflow, PTM workflow, Spider workflow, and quantitation workflow were all used. The data were refined using default settings. For the de novo search, a precursor mass tolerance of 15.0 ppm and fragment ion tolerance of 0.05 Da were employed. The enzyme was set to Trypsin, and the following modifications were added: Static Modifications – Carbamidomethylation on cysteine, TMTpro on lysine and arginine and N‐termini and Variable Modifications – acetylation on N‐termini, oxidation on methionine, and methylation on lysine and arginine residues. A maximum of 3 PTMs per peptide were allowed, and up to 5 candidates per spectrum could be reported. For the database search, a precursor mass tolerance of 15.0 ppm and fragment ion tolerance of 0.5 Da were employed. The enzyme was set to trypsin with a maximum number of missed cleavages per peptide set to 3. The data were searched against Sus scrofa protein database downloaded on September 18, 2022. The check boxes for Estimate FDR with decoy fusion, find unspecific PTMs with PEAKS PTM, and find more mutations with SPIDER were all checked. PEAKS PTM searched for the 312 built‐in modifications on de novo peptides with a de novo score greater than 15%. The quantification workflow was set for the N‐Series of TMTpro 18 plex. An error tolerance of 20.0 ppm, reporter Ions Type MS2, and FDR threshold of 1.0% were set. Data was normalized by the PEAKS software. *p*‐Values of *p* < 0.01 after correction for multiple comparisons were considered significant. ShinyGO 0.77 (South Dakota State University, Brookings, South Dakota, USA) was used for pathway analysis.

### 2.7 Immunoblotting

Immunoblotting was used to validate the results of small samples of the proteomics results. Ischemic and nonischemic myocardial tissue was lysed from eight control animals and five experimental using RIPA Lysis Buffer (# BP‐115‐5X, Boston Bioproducts, Milford, MA, USA) and Halt Protease Inhibitor Cocktail (# 87786, ThermoFisher Scientific, Waltham, MA, USA) (Sabe, Xu, et al., 2023). The lysate (40 μg) ran on a 4%–12% Bis‐Tris gel (#NP0329BOX, ThermoFisher Scientific, Waltham, MA, USA) and transferred to 0.2 μm polyvinylidene difluoride (#1620177, Bio‐Rad Laboratories, Hercules, California, USA) or nitrocellulose (#1620115, Bio‐Rad Laboratories, Hercules, California, USA). Membranes were blocked in a 5% non‐fat dry milk solution. Membranes were incubated overnight with primary antibodies dilutions. Horseradish peroxidase‐linked secondary antibodies to mouse or rabbit (Table S1, Cell Signaling, Danvers, MA) were incubated for 1 h at room temperature (Sabe, Xu, et al., 2023). Membranes were developed with ECL Western Blotting Substrate (#32106, ThermoFisher Scientific, Waltham, MA, USA) and imaged on a ChemiDoc Imaging System (Bio‐Rad, Hercules, CA, USA). Membranes were treated with Restore PLUS Western Blot Stripping Buffer (#46430, ThermoFisher Scientific, Waltham, MA, USA) and re‐blocked with 5% milk solution to allow for repeat probing. Immunoblot intensity was analyzed using NIH Image J software (Sabe, Xu, et al., 2023).

### 2.8 Antibodies

Primary antibodies to isocitrate dehydrogenase 2 (IDH 2), vinculin, succinate dehydrogenase (SDHA), fatty acid synthase (FAS), and GAPDH (Glyceraldehyde 3‐phosphate dehydrogenase) were obtained from Cell Signaling (Danvers, Massachusetts, USA). Primary antibodies to calsequestrin‐2, Troponin T, and sarcoplasmic reticulum calcium‐ATPase 1 (ATP2A1) were obtained from Proteintech (Rosemont, Illinois, USA). Primary antibody to Carnitine palmitoyltransferase I (CPT1a) and Total OXPHOS were obtained from Abcam (Cambridge, England, UK; Table S1).

### 2.9 Histochemistry

Periodic acid–Schiff (PAS) staining was performed by iHisto (iHisto, Salem, MA) on ischemic and non‐ischemic myocardial tissue from eight control and five experimental animals. Slides were analyzed using QuPath (University of Edinburgh, Edinburgh, Scotland, UK). A one‐square‐millimeter area was selected and analyzed for percentage of dense glycogen stain using QuPath automated threshold detection. (University of Edinburgh, Edinburgh, Scotland, UK).

### 2.10 Statistical analysis

Statistical analysis for raw proteomic data was described above. All other statistical analysis was performed using Prism 9 (GraphPad Software, San Diego, CA, USA). Normality was determined using Shapiro–Wilk test. Student's *t*‐test was used to analyze normal data, and non‐parametric data was analyzed with Wilcoxon rank sum. Data is presented as mean and standard deviation. Immunoblot data is mean fold change of the band intensity normalized to the average control. Outliers greater than two standard deviations from the mean were excluded. Data for the ischemic myocardial perfusion obtained in our prior study at rest and while pacing the heart at 150 beats per minute was plotted against inflammatory protein expression and analyzed using Pearson Correlation Coefficient (Sabe, Harris, et al., 2023).

## 3 RESULTS

### 3.1 Myocardial function and physical parameters

**FIGURE 1:**
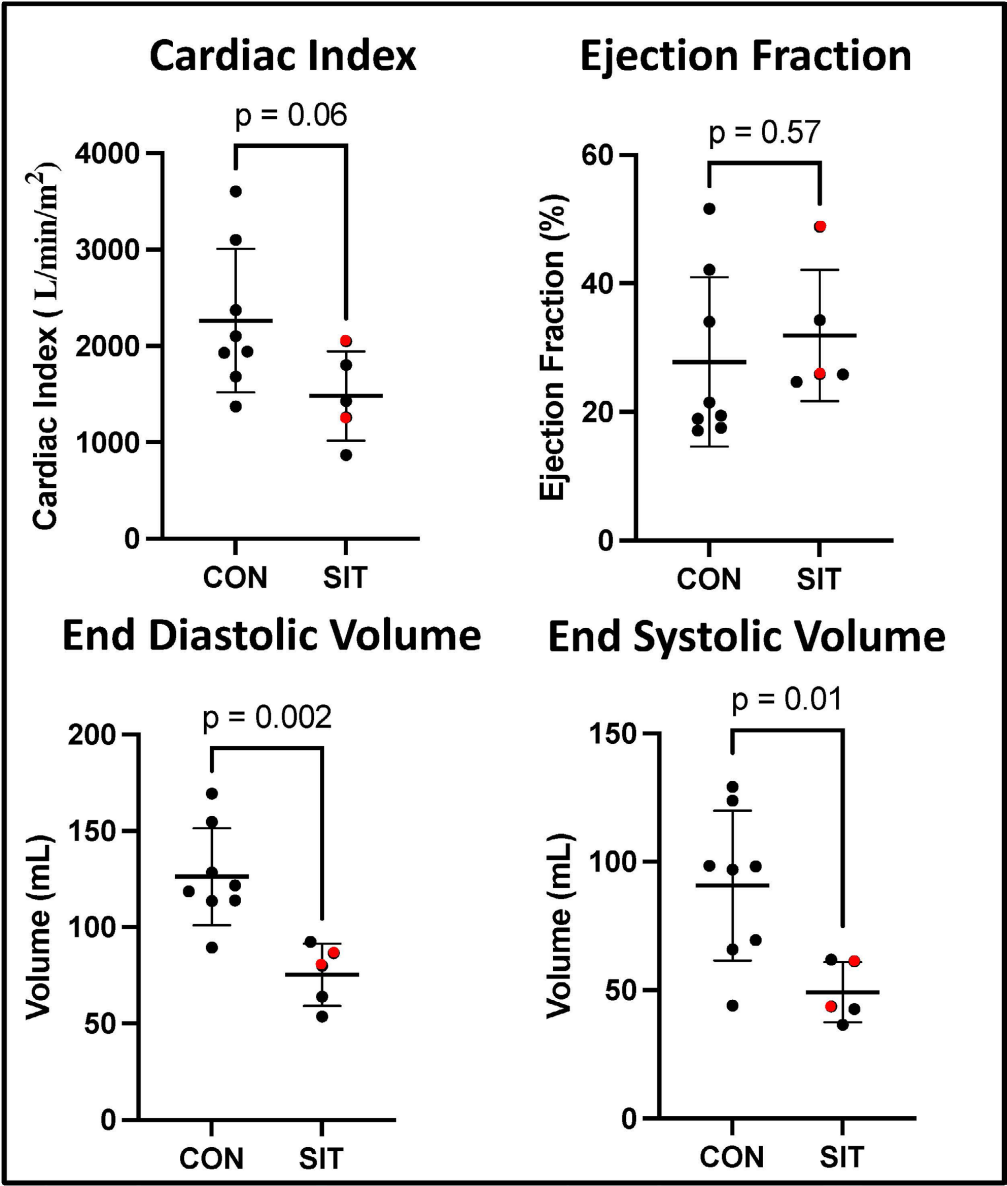
Functional data. End diastolic and systolic volume was significantly decreased in the sitagliptin (SIT) group compared to control (CON) between the two groups. There was a trend towards decreased cardiac index in the SIT group. Ejection fraction was similar between the two groups. Red dots represent the animals with interrupted treatment.

There was a significant decrease in both the end diastolic and end systolic volumes in the SIT group (*p* = 0.002, *p* = 0.01). There was a strong trend towards decreased cardiac index in the SIT group compared to CON (*p* = 0.06). There was no change in ejection fraction between the two groups (*p* = 0.57) (Figure 1). There was no difference in weight between the groups. The SIT group had a significant decrease in blood glucose 60 min after dextrose administration (*p* = 0.04, Table 1).

**TABLE 1:** Weight and glucose tolerance.

	Control	Sitsgliptin	*p*‐Value
Weight (kg, mean ± SD)	47.4 ± 7.6	55.9 ± 14.9	0.20
Starting BG Pre Dextrose (mg/dL, mean ± SD)	120.8 ± 19.7	129.2 ± 12.1	0.44
30 min BG Post Dextrose (mg/dL, mean ± SD)	263.4 ± 23.2	241.8 ± 51.9	0.37
60 min BG Post Dextrose (mg/dL, mean ± SD)	236.5 ± 26.9	197.0 ± 27.6	**0.04**

*Note*: Table 1 shows weight and glucose tolerance for the sitagliptin and control groups. Significant *p*‐values (*p* < 0.05) are highlighted in bold.

### 3.2 Proteomics and pathway analysis

**FIGURE 2:**
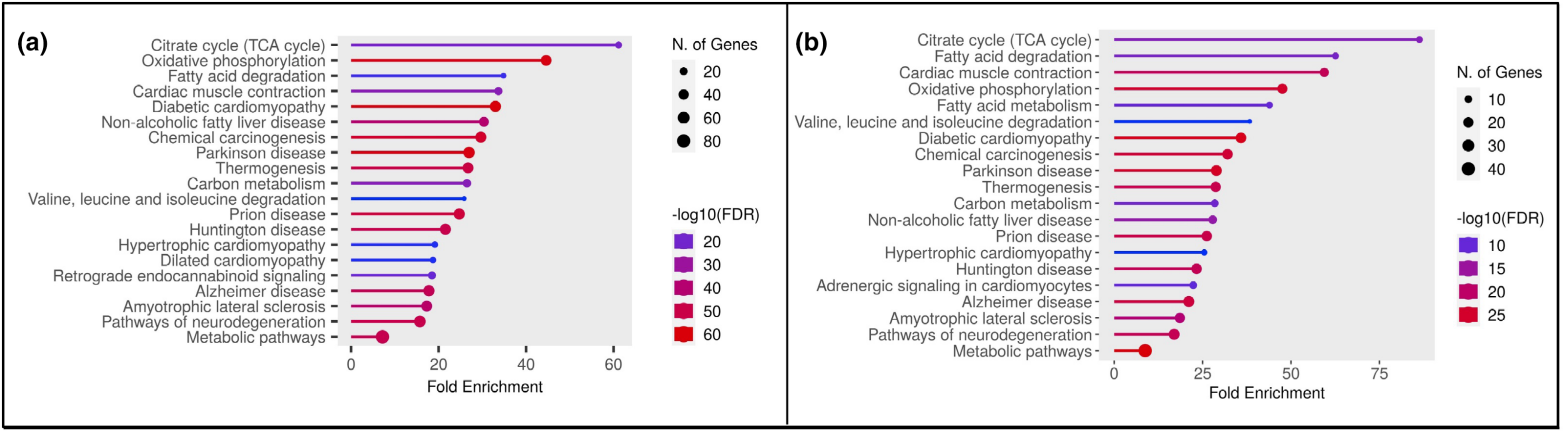
Proteomics pathway analysis. (a) Pathway analysis of decreased proteins in sitagliptin non‐ischemic myocardium compared to control. (b) Pathway analysis of decreased proteins in sitagliptin ischemic myocardium compared to control. FDR; false discovery rate.

Proteomic analysis identified 1003 total proteins in the non‐ischemic myocardium and 728 proteins in the ischemic myocardium. The two‐analysis had 590 overlapping total proteins. The non‐ischemic myocardium had 396 significantly decreased proteins in the SIT group compared to CON (all *p* < 0.01). This included proteins involved in the citric acid cycle (TCA), fatty acid oxidation (FAO), calcium handling, myocardial contractility, and oxidative phosphorylation (OXPHOS) (Figure 2a). The ischemic myocardium had 166 significantly decreased proteins in the SIT group (all *p* < 0.01). This included proteins involved in OXPHOS, calcium handling, myocardial contractility, the TCA, and FAO (Figure 2b). The significantly decreased proteins had 151 overlapping total proteins between the ischemic and non‐ischemic comparison. There were no significantly increased proteins in the ischemic or non‐ischemic myocardium between the SIT and CON groups.

### 3.3 The citric acid cycle, fatty acid oxidation, and glycogen metabolism

**FIGURE 3:**
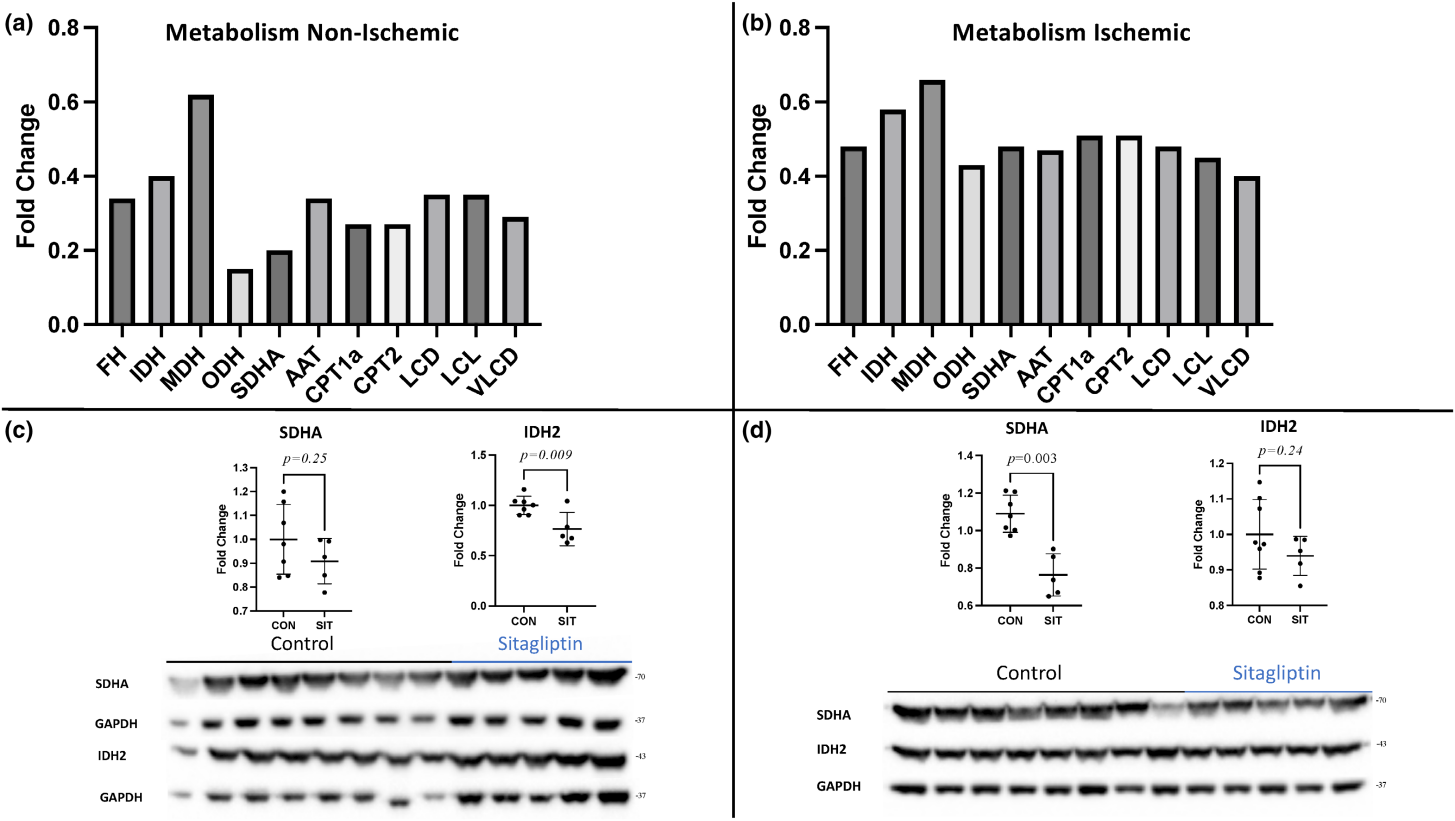
The citric acid cycle and fatty acid oxidation. (a) The changes in enzymes in the citric acid cycle (TCA) and fatty acid oxidation including fumarate hydratase (FH), isocitrate dehydrogenase (IDH), malate dehydrogenase (MDH), 2‐oxoglutarate dehydrogenase (ODH), succinate dehydrogenase (SDA), acetyl‐CoA acetyltransferase (AAT), carnitine O‐palmitoyltransferase 2 (CPT1a), carnitine O‐palmitoyltransferase 2 (CPT2), long‐chain 3‐hydroxyacyl‐CoA dehydrogenase (LCD), long‐chain‐fatty‐acid‐CoA ligase (LCL), and very long chain acyl‐CoA dehydrogenase (VLCD) between non‐ischemic sitagliptin (SIT) and control (CON) represented as fold change (all *p* < 0.01). (b) The changes in enzymes in the TCA cycle and FAO including FH, IDH, MDH, ODH, SDA, AAT, CPT1a, CPT2, LCD, and VLCD between ischemic SIT and control (CON) represented as fold change (all *p* < 0.01). LCL was trending towards a decrease in the ischemic SIT compared to control (*p* = 0.38). (c) Immunoblotting validation of pathway related protein changes in non‐ischemic SIT compared to non‐ischemic CON. (d) Immunoblotting validation of validation of pathway related protein changes in ischemic SIT compared to ischemic CON.

Pathway analysis identified the TCA and FAO as key pathways decreased in both the non‐ischemic and ischemic myocardium of the SIT group. In the SIT‐N myocardium, we found decreases in TCA enzymes including fumarate hydratase, IDH 1, IDH 2 malate dehydrogenase, 2‐oxoglutarate dehydrogenase, and SDA (all *p* < 0.01). The SIT‐N myocardium also had a decrease in enzymes involved in FAO including acetyl‐CoA acetyltransferase, CPT1a, carnitine O‐palmitoyltransferase 2, long‐chain 3‐hydroxyacyl‐CoA dehydrogenase, long‐chain‐fatty‐acid‐CoA ligase, and very long‐chain acyl‐CoA dehydrogenase (all *p* < 0.01) (Figure 3a). The SIT‐I myocardium also had decreases in the same TCA enzymes including fumarate hydratase, IDH 1, IDH 2, malate dehydrogenase, 2‐oxoglutarate dehydrogenase, and SDHA (all *p* < 0.01). The SIT‐I myocardium also had a decrease in similar enzymes involved in fatty acid oxidation including acetyl‐CoA acetyltransferase, CPT1a, carnitine O‐palmitoyltransferase 2, long‐chain‐fatty acid‐CoA ligase, and very long chain acyl‐CoA dehydrogenase (all *p* < 0.01; Figure 3b). Immunoblot validation for metabolic markers confirmed the decreases in IDH 2 in SIT‐N compared to CON‐N (*p* = 0.009; Figure 3c). SDHA was significantly decreased in SIT‐I compared to CON‐I (*p* = 0.003; Figure 3d).

**FIGURE 4:**
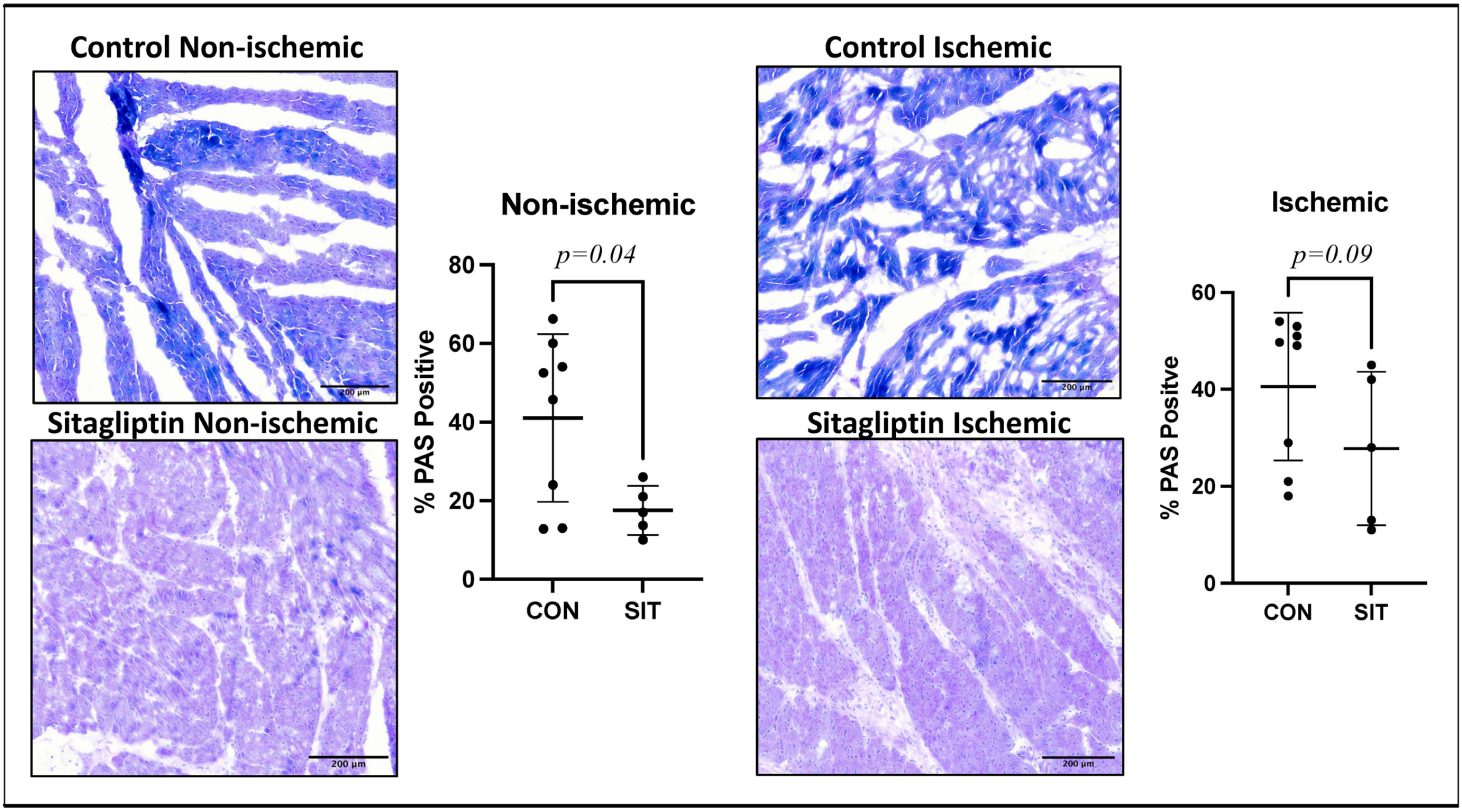
Periodic acid–Schiff stain. Periodic acid–Schiff stain (PAS) staining showed a significant decrease in glycogen content in the non‐ischemic sitagliptin (SIT) group compared to control (CON), and a strong trend towards decreased glycogen content in the ischemic SIT compared to CON.

Glycogen phosphorylase expression was decreased on the proteomics data for both the SIT‐N and SIT‐I group. PAS staining showed a significant decrease in glycogen content in the SIT‐N compared to CON‐N (*p* = 0.04) and a strong trend towards decreased glycogen content in the SIT‐I compared to CON‐I (*p* = 0.09; Figure 4).

### 3.4 Calcium handling and myocardial contractility

**FIGURE 5:**
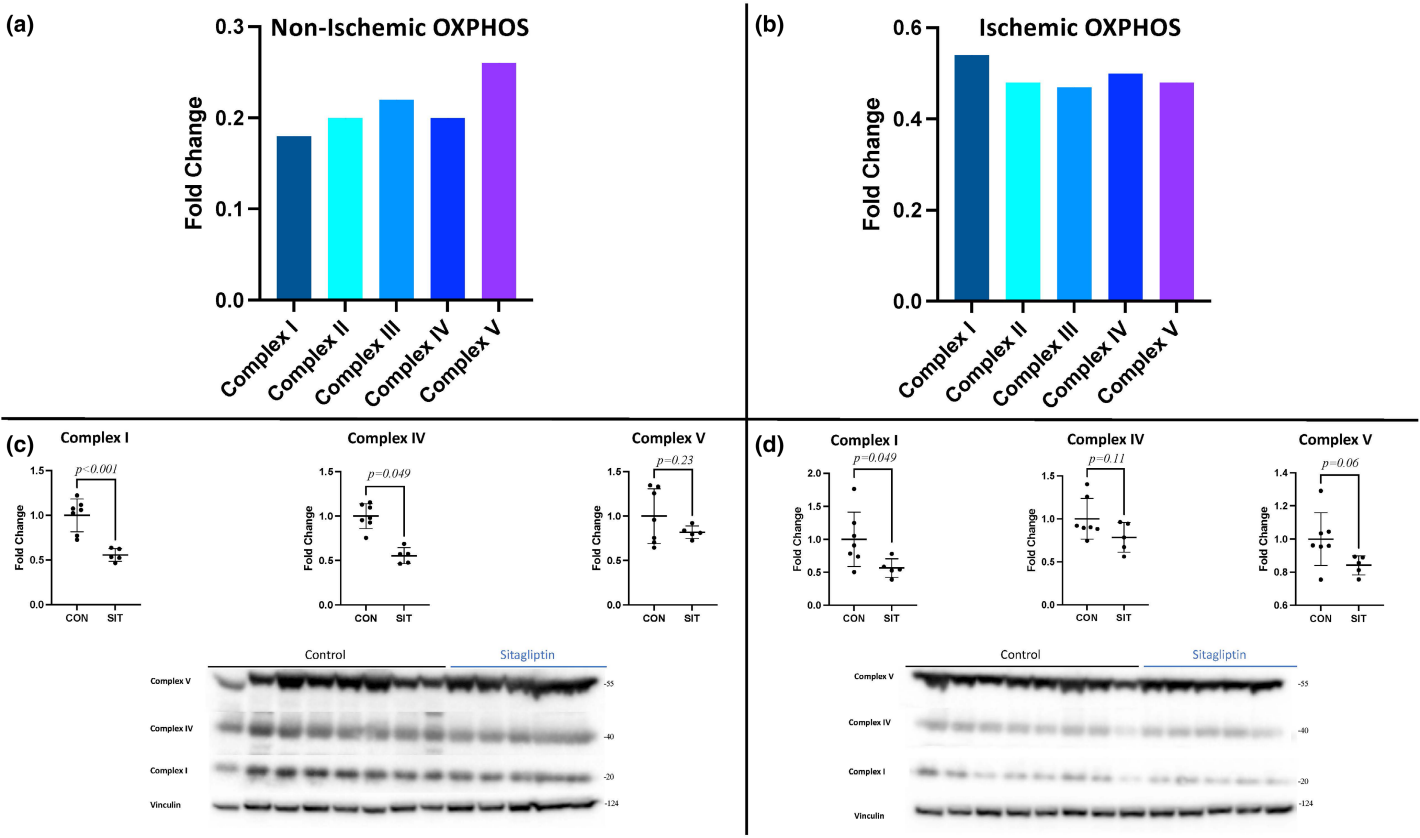
Oxidative phosphorylation. (a) The changes in mitochondrial complexes 1–V between non‐ischemic sitagliptin (SIT) and control (CON) represented as fold change (all *p* < 0.01). (b) The changes in mitochondrial complexes 1‐V between ischemic SIT and control CON represented as fold change (all *p* < 0.01). (c) Immunoblotting validation of mitochondrial complex changes in non‐ischemic SIT compared to non‐ischemic CON. (d) Immunoblotting validation of mitochondrial complex changes in ischemic SIT compared to ischemic CON. Oxidative phosphorylation, OXPHOS.

Pathway analysis identified several decreased proteins involved in myocardial calcium handling and contractility in both the non‐ischemic and ischemic myocardium of the SIT group. The SIT‐N myocardium had decreased expression of proteins involved in myocardial contraction inducing troponin T, troponin C, troponin I, and myosin light chain 3, and decreases in important myocardial calcium‐regulating proteins inducing calsequestrin‐2, voltage‐dependent calcium channel, and sarcoplasmic reticulum calcium ATPase 1 (all *p* < 0.01) (Figure 5a). The SIT‐I myocardium had decreased expression of proteins involved in myocardial contraction inducing troponin T, troponin C, and myosin light chain 3, and decreases in important myocardial calcium‐regulating proteins inducing calsequestrin‐2, voltage‐dependent calcium channel, and sarcoplasmic reticulum calcium ATPase 1 (all *p* < 0.01) (Figure 5b). Troponin I was trending towards a decrease in the SIT‐I tissue (*p* = 0.01). Immunoblot validation for metabolic markers confirmed the decreases in calsequestrin‐2, Troponin T, and ATP2A1 in both ischemic and non‐ischemic groups (all *p* < 0.05; Figure 5c,d).

### 3.5 Oxidative phosphorylation

**FIGURE 6:**
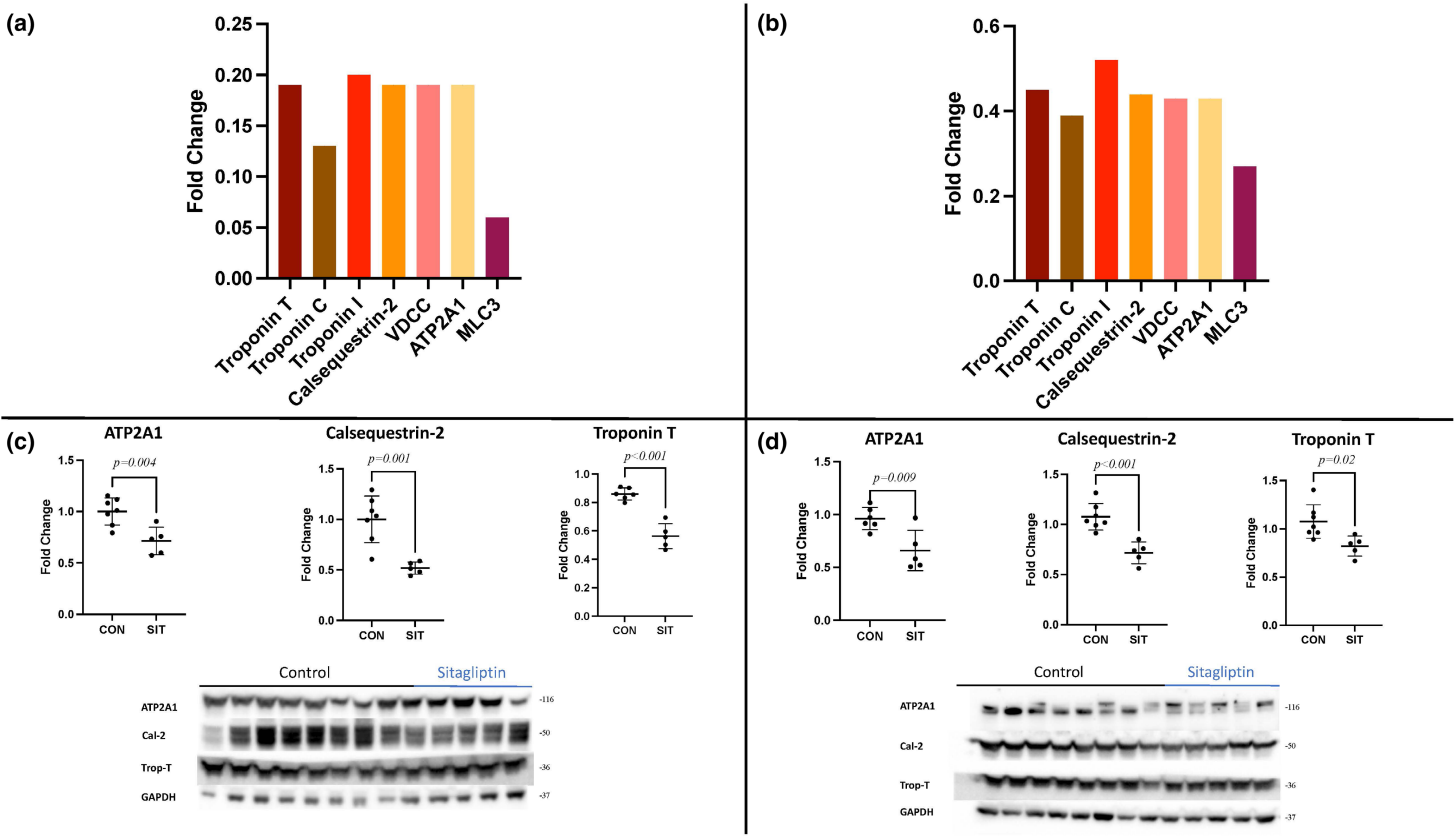
Calcium handling and myocardial contractility. (a) The changes in calcium handling and myocardial contractility including troponin I, troponin C, troponin T, calsequestrin‐2 (Cal‐2), voltage dependent calcium channels (VDCC), sarcoplasmic reticulum calcium‐ATPase 1 (ATP2A), and myosin light chain 3 (MLC3) between non‐ischemic sitagliptin (SIT) and control (CON) represented as fold change (all *p* < 0.01). (b) The changes in calcium handling and myocardial contractility including troponin C, troponin T, calsequestrin‐2 (Cal‐2), VDCC, ATP2A, and MLC3 between ischemic sitagliptin (SIT) and control (CON) represented as fold change (all *p* < 0.01). Troponin I had a strong trend towards a decrease *p* = 0.01. (c) Immunoblotting validation of pathway related protein changes in non‐ischemic SIT compared to non‐ischemic CON. (d) Immunoblotting validation of validation of pathway related protein changes in ischemic SIT compared to ischemic CON.

Pathway analysis identified several mitochondrial complex and complex subunits as being decreased in both the non‐ischemic and ischemic SIT‐treated myocardium. The SIT‐N myocardium had decreased expression of Complex I, Complex II, Complex III, Complex IV, and Complex V (all *p* < 0.01) (Figure 6a). The SIT‐I myocardium had decreased expression of Complex I, Complex II, Complex III, Complex IV, and Complex V (all *p* < 0.01; Figure 6b). Immunoblot validation confirmed proteomic results in the SIT‐N showing decreases in complex I and complex IV (all *p* < 0.05) with a trend to decreasing complex V (*p* = 0.23; Figure 6c). Immunoblot validation in the SIT‐I showed significant decreases in Complex I (*p* = 0.049) and trends towards decreased complex IV and V (*p* = 0.11, *p* = 0.06).

### 3.6 Myocardial perfusion protein correlation

Myocardial perfusion data from the ischemic myocardium obtained in our prior study was plotted against protein expression (Sabe, Harris, et al., 2023). There was a significant inverse relationship between FAS expression in the non‐ischemic myocardium and myocardial perfusion (*p* = 0.02). There was a correlation between sarcoplasmic reticulum calcium ATPase, calsequestrin‐2, and Troponin T expression in the non‐ischemic myocardium and myocardial perfusion (all *p* < 0.05). There was no other statistically significant correlation between myocardial perfusion and protein expression with any of the Immunoblot validated markers tested (Table 2).

**TABLE 2:** Myocardial perfusion protein correlation.

	Non‐ischemic rest		Non‐ischemic paced		Ischemic rest		Ischemic paced	
	*r*	*p*	*r*	*p*	*r*	*p*	*r*	*p*
Metabolism								
Carnitine palmitoyltransferase I	0.57	0.31	0.41	0.49	−0.56	0.33	−0.32	0.60
Fatty Acid Synthase	−0.93	**0.02**	−0.14	0.83	0.12	0.84	0.06	0.92
Isocitrate dehydrogenase 2	0.71	0.18	0.01	0.98	−0.05	0.94	0.57	0.32
Succinate dehydrogenase	0.54	0.34	−0.33	0.59	0.50	0.39	0.44	0.46
Mitochondria complexes								
Complex I	0.02	0.97	0.67	0.22	0.73	0.16	−0.08	0.90
Complex II	0.17	0.78	0.65	0.23	0.54	0.35	−0.03	0.97
Complex IV	0.02	0.97	0.67	0.22	0.09	0.88	−0.35	0.56
Complex V	0.19	0.75	0.58	0.30	0.34	0.57	0.18	0.76
Contraction								
Sarcoplasmic reticulum calcium‐ATPase 1	0.93	**0.02**	0.03	0.96	−0.31	0.61	0.67	0.21
Calsequestrin‐2	0.97	**0.01**	−0.17	0.78	−0.11	0.86	0.39	0.51
Troponin T	0.88	**0.05**	−0.04	0.95	−0.30	0.62	0.32	0.59

*Note*: Table 1 shows the correlation between protein expression and myocardial perfusion at rest and while pacing the heart at 150 beats per minute in both the ischemic and non‐ischemic tissue. Correlation coefficient (*r*) was calculated using Pearson's test. Significant *p*‐values (*p* < 0.05) are highlighted in bold.

## 4 DISCUSSION

In human studies, DPP‐4 inhibitors have shown mixed results with most studies showing no benefit to cardiovascular outcomes from the use of DPP‐4 inhibitors (Patoulias et al., 2021). We believe that functional data and pathways discovered in our proteomic analysis using large animal model could help explain some of the negative effects of DPP‐4 inhibitors seen in clinical studies. Our previously published study was complicated by a mortality rate of 50% in the SIT group (Sabe, Harris, et al., 2023). One swine was euthanized for symptoms of congestive heart failure, and the other four swine died late sudden deaths likely due to an arrhythmia. The surviving swine in the SIT group had increased coronary perfusion; however, there was a trend towards decreased cardiac output and decreased stroke volume (Sabe, Harris, et al., 2023). In the current study, further analysis of our functional data showed a significant decrease in the end diastolic and end systolic volume and a very strong trend towards decreased cardiac index. There was notably no change in ejection fraction. The decrease in end diastolic and end systolic volume along with a trend towards decreased cardiac index would be similar to the findings seen in an early hypertrophic cardiomyopathy. The findings reported here could also explain the preserved ejection fraction but overall trend towards decreased cardiac function.

Healthy myocardial tissue derives much of its energy from fatty acid oxidation, and the metabolic changes associated with myocardial ischemia have been well described (Lopaschuk et al., 2010). Myocardial ischemia results in a dysregulation of myocardial metabolism (Lopaschuk et al., 2010). Dysregulation can take the form of increased fatty acid oxidation, as is seen in CHF, or decreased FAO and increased glycolysis, as is seen in myocardial ischemia. The data from our study showed a significant decrease in several key enzymes in the TCA including fumarate hydratase, IDH 1, IDH 2, malate dehydrogenase, 2‐oxoglutarate dehydrogenase, and succinate dehydrogenase in both the SIT‐N and SIT‐I myocardium compared to CON‐N and CON‐I (Haddad & Mohiuddin, 2023). This would imply that sitagliptin is resulting in a decrease in the utilization of the TCA shunting energy production to other pathways.

Glycogen is an important energy source during myocardial ischemia (Lopaschuk & Stanley, 1997). Both SIT‐N and SIT‐I myocardium had a significant decrease in glycogen phosphorylase. This could imply a decrease in the utilization of glycolysis to fuel myocardial energy production. More interestingly, there was a significant decrease in total glycogen in the SIT‐N compared to CON‐N and a strong trend towards decreased glycogen in the SIT‐I compared to CON‐I groups. This implies a global decrease in myocardial glycogen stores associated with sitagliptin treatment.

The SIT‐N and SIT‐I myocardium also had decreases in enzymes involved in FAO including acetyl‐CoA acetyltransferase, CPT1a, carnitine O‐palmitoyltransferase 2, long‐chain‐fatty acid CoA ligase, and very long‐chain acyl‐CoA dehydrogenase compared to CON‐N and CON‐I (Talley & Mohiuddin, 2023). The decrease in key enzymes of fatty acid oxidation particularly CPT1a would suggest that the global SIT myocardium has a decrease in FAO. The decrease in both FAO and TCA in both the SIT‐N and SIT‐I myocardium would imply that the SIT‐treated myocardium has an increased reliance on glycolysis for energy production. This is concerning as healthy myocardial tissue derives the majority of its energy from fatty acid oxidation, and sitagliptin is resulting in a dysregulation of normal myocardial metabolism (Lopaschuk et al., 2010).

Healthy cardiomyocytes utilize the energy substates obtained from FAO to fuel myocardial OXPHOS (Lopaschuk et al., 2021). As one would expect with decreased FAO and TCA, our study revealed a significant decrease in mitochondrial OXPHOS. The SIT‐N and SIT‐I groups had decreases in mitochondrial Complexes I–V in SIT‐N and SIT‐I compared to CON‐N and CON‐I. This is fitting with a shift towards more primitive energy production in the myocardium such as to glycolysis, and again represents a global decrease in normal myocardial metabolism with SIT treatment.

The myocardium utilizes the energy produced to drive myocardial contraction. Myocardial contraction utilizes a complex interplay of myocardial structural proteins and calcium signaling. SIT‐N and SIT‐I tissue both had decreases in important contractile proteins including troponin T, troponin C, and myosin light chain 3 compared to CON‐N and CON‐I (Adamcová & Pelouch, 2003; Léger et al., 1975). The decrease in contractile protein was accompanied by decreases in important myocardial calcium‐regulating proteins including calsequestrin‐2, voltage‐dependent calcium channel, and sarcoplasmic reticulum calcium ATPase 1 in both the SIT‐N and SIT‐I myocardium (Lou et al., 2012). This likely represents a global decrease in myocardial calcium‐dependent contractility. This decrease in contractility could help explain the trends towards decreased cardiac output and cardiac index seen in the SIT group.

In summary, our study showed a significant decrease in the end diastolic and end systolic volume in the SIT group and a strong trend towards decreased cardiac index, similar to findings seen in hypertrophic cardiomyopathy. The functional changes were accompanied by decreases in proteins involved in the TCA, glycogen breakdown, FAO, OXPHOS, calcium handing, and myocardial contraction in both SIT‐N and SIT‐I myocardium. The decrease in in protein expression is mostly independent of coronary perfusion. These results plausibly represent a global decrease in myocardial metabolism with SIT treatment, and this global decrease in myocardial metabolism could be linked to the global decrease in calcium handing and myocardial contraction proteins in the SIT group. The results of our study shed light on the potentially negative effects of SIT on myocardial function, metabolism, calcium handing, and contraction. These findings could help explain the previously reported high mortality in the sitagliptin group (Sabe, Harris, et al., 2023). This study furthers our understanding of how DPP‐4 inhibitors could exacerbate heart failure and provides targets for further investigation in humans.

This study is not without limitations. It is limited by sample size due to the high mortality in the SIT group and the nature of large animal work. The pause in treatment of two SIT animals by the IACUC could result in skewed results as only three animals completed the whole protocol. The protein distribution in the proteomics appeared similar among the SIT individuals with limited treatment and complete treatment, but this is limited by the number of proteins detected. We attempted to use a mix of males and females in the study, but the SIT group only had one surviving female. This makes the study underpowered to evaluate sex‐specific differences. The pigs in this study are sexually immature, and as a result, the study might not account for interactions with sex hormones. The animals in this study are otherwise healthy without many common comorbid conditions of CAD patients including metabolic syndrome, smoking, and polypharmacy. As such, our model might miss some complex interactions seen in humans. Finally, this study is limited by the specific proteomic techniques used in it. Different proteomic techniques might identify more proteins, and the techniques used in the study do not allow for the cross‐comparison of the ischemic and non‐ischemic groups.

## 5 CONCLUSIONS

SIT treatment was associated with a decrease in the end diastolic and end systolic volumes and a strong trend towards decreased cardiac index in the SIT group, similar to finding seen in hypertrophic cardiomyopathy. SIT treatment was also associated with a global decrease in expression of proteins involved in the TCA, FAO, OSPHOS, calcium handing, and myocardial contraction in both SIT‐N and SIT‐I myocardium. These findings help expand our understanding of how DPP‐4 inhibitor exacerbate heart failure and provide targets for further investigation in humans; however, it is important to acknowledge the limited sample size due to mortality in the study.

## FUNDING INFORMATION

This research was funded by NIH T32HL160517 (D.D.H., M.B.); the National Heart, Lung, and Blood Institute (NHLBI) 1F32HL160063–01 (S.A.S.); T32 GM065085 (C.M.X.); R01HL133624 and R56HL133624–05 (M.R.A.); R01HL46716 and R01HL128831 (F.W.S.).

## CONFLICT OF INTEREST STATEMENT

The authors have no conflicts of interest to disclose.

## ETHICS STATEMENT

The protocol was approved by the Rhode Island Hospital Institutional Animal Care and Use Committee (IACUC) (Protocol #505821). Animals were cared for in compliance with the Principles of Laboratory Animal Care and the Guide for the Care and Use of Laboratory Animals.

## Supporting information


Table S1.

## Data Availability

All data are alliable by request from the corresponding author.
