# Supplementary material for: DPP‐4 inhibitor sitagliptin treatment results in altered myocardial metabolic proteome and oxidative phosphorylation in a swine model of chronic myocardial ischemia
Source: Physiol Rep. 2024 Mar 12;12(5):e15976. doi: 10.14814/phy2.15976 (PMC10933084; doi:10.14814/phy2.15976)
Supplement: Supplementary file 1 — Table S1. [file PHY2-12-e15976-s001.docx]

| **Antibody Name** | **Dilution** | **Company** | **Catalog Number** |
| --- | --- | --- | --- |
|  |  |  |  |
| Carnitine palmitoyltransferase I (CPT1a) | 1:1000 | abcam | ab128568 |
| Total OXPHOS | 1:1000 | abcam | ab110413 |
| Calsequestrin 2 | 1:1000 | Proteintech | 18422-1-AP |
| Cardiac Troponin T | 1:1000 | Proteintech | 15513-1-AP |
| SERCA2 | 1:1000 | Proteintech | 13985-1-AP |
| Isocitrate Dehydrogenase 2 | 1:1000 | Cell Signaling | 56439 |
| Vinculin | 1:1000 | Cell Signaling | 13901 |
| Succinate Dehydrogenase | 1:1000 | Cell Signaling | 11998 |
| Fatty Acid Synthase | 1:1000 | Cell Signaling | 3189 |
| GAPDH | 1:1000 | Cell Signaling | 97166 |
| Mouse Secondary Antibody | 1:4000 | Cell Signaling | 7076 |
| Rabbit Secondary Antibody | 1:4000 | Cell Signaling | 7074 |

**Supplemental table 1. Antibodies:** Supplemental Table one shows the primary and secondary antibodies with catalog number and dilution.
